# A novel monoclonal antibody generated by immunization with granular tau oligomers binds to tau aggregates at 423-430 amino acid sequence

**DOI:** 10.1038/s41598-024-65949-7

**Published:** 2024-07-26

**Authors:** Yoshiyuki Soeda, Emi Hayashi, Naoko Nakatani, Shinsuke Ishigaki, Yuta Takaichi, Taro Tachibana, Yuichi Riku, James K. Chambers, Riki Koike, Moniruzzaman Mohammad, Akihiko Takashima

**Affiliations:** 1 Laboratory for Alzheimer’s Disease, Department of Life Science, Faculty of Science, Gakushuin University, 1-5-1 Mejiro, Toshima-ku, Tokyo, 171-8588 Japan; 2 Cell Engineering Corporation, 5-12-14 Nishinakajima, Yodogawa-ku, Osaka, 532-0011 Japan; 3 Department of Diagnostics and Therapeutics for Brain Disease, Molecular Neuroscience Research Center, Shiga University of Medical Science, Seta Tsukinowa-cho, Otsu, Shiga 520-2192 Japan; 4 Laboratory of Veterinary Pathology, Graduate School of Agricultural and Life Sciences, The University of Tokyo, 1-1-1 Yayoi, Bunkyo-ku, Tokyo, 113-8657 Japan; 5 Graduate School of Engineering Division of Science and Engineering for Materials, Chemistry and Biology, Osaka Metropolitan University, 3-3-138 Sugimoto, Sumiyoshi-ku, Osaka-shi, Osaka, 558-0022 Japan; 6 Institute for Medical Science of Aging, Aichi Medical University, 1-1 Yazakokarimata, Nagakute, Aichi 480-1195 Japan; 7 Department of Neurology, Nagoya University, 65 Tsurumai, Showa, Nagoya, Aichi 466-8550 Japan

**Keywords:** Tau aggregation, Granular tau oligomers, Antibody, C-terminal regions of tau, Alzheimer's disease, Protein aggregation

## Abstract

Prior to the formation of amyloid fibrils, the pathological hallmark in tau-related neurodegenerative disease, tau monomers aggregate into a diverse range of oligomers. Granular tau oligomers, consisting of approximately 40 tau protein molecules, are present in the prefrontal cortex of patients at Braak stages I-II, preclinical stages of Alzheimer’s disease (AD). Antibodies to granular tau oligomers as antigens have not been reported. Therefore, we generated new rat monoclonal antibodies by immunization with granular tau oligomers. Three antibodies from different hybridoma clones showed stronger immunoreactivity to granular tau oligomers and tau fibrils compared with monomeric tau. Of the three antibodies, 2D6-2C6 showed 3000-fold greater immunoreactivity in P301L-tau transgenic (rTg4510) mice than in non-transgenic mice, while MC1 antibody, which detects pathological conformations of tau, showed a 5.5-fold increase. These results suggest that 2D6-2C6 recognizes aggregates more specifically than MC1. In AD subjects, 2D6-2C6 recognized neurofibrillary tangles and pretangles, and co-localized within AT8-positive cells containing phosphorylated tau aggregates. The epitope of 2D6-2C6 is the 423–430 amino acid (AA) sequence of C-terminal regions. Taken together, a novel monoclonal antibody, 2D6-2C6, generated by immunization with granular tau oligomers binds to tau aggregates at the 423–430 AA sequence.

## Introduction

Tau is a microtubule-associated protein that is highly soluble in healthy neurons. Neurofibrillary tangles (NFTs), a pathological hallmark of Alzheimer’s disease (AD), are composed primarily of hyperphosphorylated and insoluble aggregated forms of tau^1^. Brains of individuals with neurodegenerative tauopathies, including Pick’s disease, chronic traumatic encephalopathy, corticobasal degeneration, and progressive supranuclear palsy, show abnormal tau accumulation. In addition, tau aggregation correlates with dementia severity and neuronal loss in AD^2–4^. Mutations in the microtubule-associated protein tau (MAPT) gene are responsible for frontotemporal dementia with parkinsonism linked to chromosome 17 (FTDP-17), a hereditary neurodegenerative tauopathy. Many reports have correlated tau pathological changes and neuronal atrophy in transgenic mice harboring single mutant tau^5,6^. The prion-like propagation model of tau proposes that extracellular tau seeds released from neurons are responsible for the propagation of tau pathology in cell models and in vivo^7–9^. These findings have paved the way for the development of biologics, including antibody drugs targeting tau. Furthermore, fifteen drugs, including active and passive immunotherapies targeting tau, have undergone clinical trials for AD and related tauopathies^10^.

Tau-related toxicity, such as neuronal loss and behavioral abnormalities, is involved in tau oligomer formation but not in monomeric tau and NFT, according to many previous studies^6,11–13^. Tau pathology and tau-related toxicity are induced by extracellular, intracellular or both types of tau oligomers^13–15^. Exposure to recombinant tau oligomers prepared by polymerization reactions impairs synaptic plasticity and causes neurotoxicity^16,17^. Extracellular tau oligomers have been observed in vesicles from the brains of AD patients, and these vesicular tau oligomers propagate tau pathology in vivo^18^. These previous papers indicate that the extracellular tau oligomer released from cells bearing abnormal aggregated tau is a candidate for the tau seed, which propagates tau pathology. Therefore, visualization tools to detect oligomeric tau are useful for elucidating the disease mechanism of tauopathies. However, the development of tools to detect tau oligomers is insufficient.

We identified granular tau oligomers preceding NFT formation in an in vitro study^19^. Granular tau oligomers are 15–25 nm sarkosyl-insoluble aggregates comprising approximately 40 tau proteins^19^. Unlike soluble oligomers, granular oligomer structures can be detected by atomic force microscopy (AFM)^19^. In vivo experiments have shown that granular tau oligomers contribute to neuronal cell death^5,20–22^. Furthermore, a study using human tissue found increased levels of granular tau oligomers in the frontal cortex from Braak stage I-II subjects^23^. These results suggest that granular tau oligomers may be an early marker of AD and a target for early intervention. However, no antibodies against granular tau oligomers have been reported. In this study, we developed antibodies against granular tau oligomers using an iliac lymph node method^24,25^, and analyzed the binding of the antibodies to tau aggregates in vitro and in vivo.

## Results

### Screening of antibodies derived from rat immunized granular tau oligomers

We utilized the iliac lymph node method^24,25^ to produce rat monoclonal antibodies targeting granular tau oligomers. This method has been demonstrated to yield approximately 10 times more positive hybridomas than conventional techniques that employ mouse or rat spleen cells^26^. We collected culture media from 768 hybridoma colonies (Supplemental Fig. 1) and assessed the immunoreactivity of the antibodies using ELISA and dot blot assays with immobilized recombinant granular tau oligomers. Three hybridomas (2B2, 2D6, and 8D6) exhibited strong granular tau oligomer immunoreactivity Supplemental Fig. 1). Subsequently, single cells were isolated and cloned from these three hybridomas. The cultured media obtained from 573 of these cloned colonies underwent ELISA and dot blot analyses (Supplemental Fig. 1). Through this process, we successfully identified three specific antibodies (2B2-1B6, 2D6-2C6, and 8D6-1F7) that exhibit binding affinity to granular tau oligomers (Supplemental Fig. 1).

**Figure 1:**
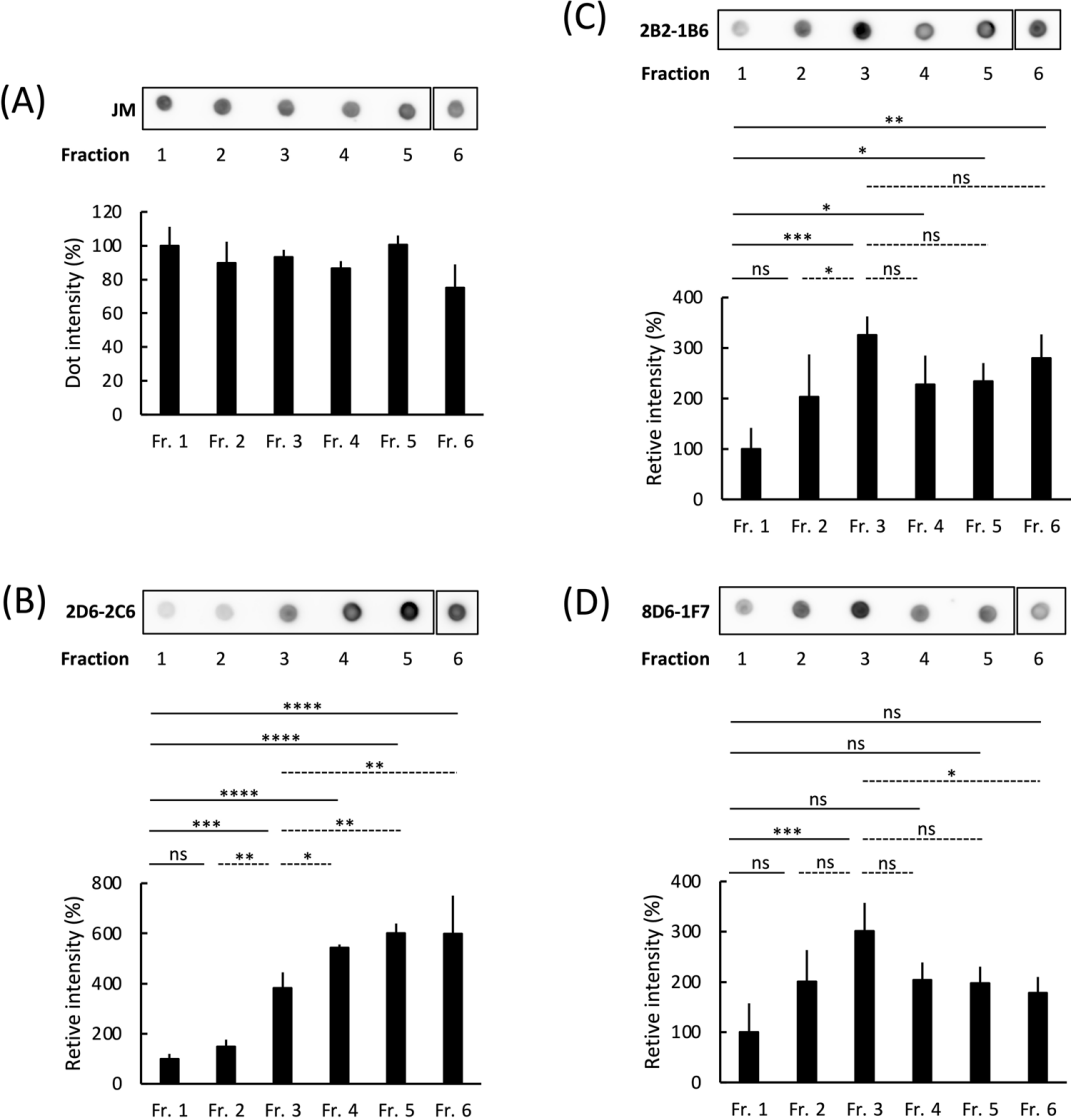
Binding of antibodies against granular tau oligomers to fractionated recombinant tau aggregates. Recombinant human full-length (FL) tau was polymerized by heparin and then fractionated into Fractions (Fr) 1–6 through sucrose step gradient centrifugation. As the fraction number increases, denser tau aggregates are observed ^19^. For instance, Fr. 1 contains monomeric and multimeric tau, while granular tau oligomers are predominantly found in Fr. 3. Fibrils are present in Fr. 4–6. Dot blot analysis detected tau in Fr. 1–6 using the pan-tau rabbit polyclonal antibody JM (**A**) and monoclonal antibodies such as 2D6-2C6 (**B**), 2B2-1B6 (**C**) and 8D6-1F7 (**D**), derived from rat immunized with granular tau oligomers. Fraction 1 contained tau at a concentration of 100 μg/ml. Tau immunoreactivity was quantified by densitometry (ImageJ software), and the levels of 2D6-2C6, 2B2-1B6 and 8D6-1F7-reactive tau were normalized to the corresponding JM-reactive tau. Quantitative data are presented as a percentage of Fr. 1 (mean ± SD from 4 experiments). P values were determined using one-way ANOVA followed by Tukey’s multiple comparisons test. Significance levels are indicated as *(p < 0.05), **(p < 0.01), ***(p < 0.001), ****(p < 0.0001), and ns denotes not significant. There are annotations on solid lines (comparing Fr. 1 to Fr. 2, 3, 4, 5 or 6) and dotted lines (comparing Fr. 3 to Fr. 2, 4, 5 or 6). The dotted signals of Fr. 6 were cropped from different parts of the same membrane (Supplemental Fig. 5A–D).

During tau aggregation, monomeric tau polymerizes, eventually forming fibrillar tau through intermediate aggregates called tau oligomers and granular tau oligomers^19^. Sucrose density gradient centrifugation can separate aggregated tau samples from recombinant tau (Supplemental Fig. 4)^19^ and brain tissue^23,27^ into six fractions based on their aggregate size and density. Each of the six fractions consists of the following tau: Fraction 1, no apparent aggregates; Fraction 2, small tau granules; Fraction 3, granular tau oligomers; and Fractions 4–6, short and long tau fibrils (Supplemental Fig. 4)^19^. We assessed the binding efficacy of our three antibodies with these tau fractions using dot blot analysis. The control pan-tau antibody (JM; derived from immunogen 2N4R-tau)^28^ showed similar immunoreactive intensities for tau across all fractions (Fig. 1A). The 2D6-2C6 antibody showed a 3.9-, 5.4-, and 6.0-fold increase in immunoreactivity in Fractions 3, 4, and 5–6, respectively, compared with Fraction 1 (monomer) (Fig. 1B). The 2B2-1B6 antibody showed increased immunoreactivity in Fractions 2 (2.0-fold), 3 (3.3-fold), 4 (2.3-fold), 5 (2.3-fold), and 6 (2.8-fold) compared with Fraction 1 (Fig. 1C). The 8D6-1F7 antibody immunoreactivity of Fractions 2, 3, 4, 5 and 6 was 2.0-, 3.0-, 2.0-, 2.0-, and 1.8-fold higher than that of Fraction 1, respectively (Fig. 1D). Similar results were observed in experiments using the three antibodies at different tau concentrations (Supplemental Fig. 2). Together, these results show that 2D6-2C6 is immunoreactive for granular tau oligomers and fibrils, while 2B2-1B6 and 8D6-1F7 bind smaller granular tau to tau fibrils.

### In vivo immunoreactivity of 2D6-2C6, 2B2-1B6, and 8D6-1F7

**Figure 2:**
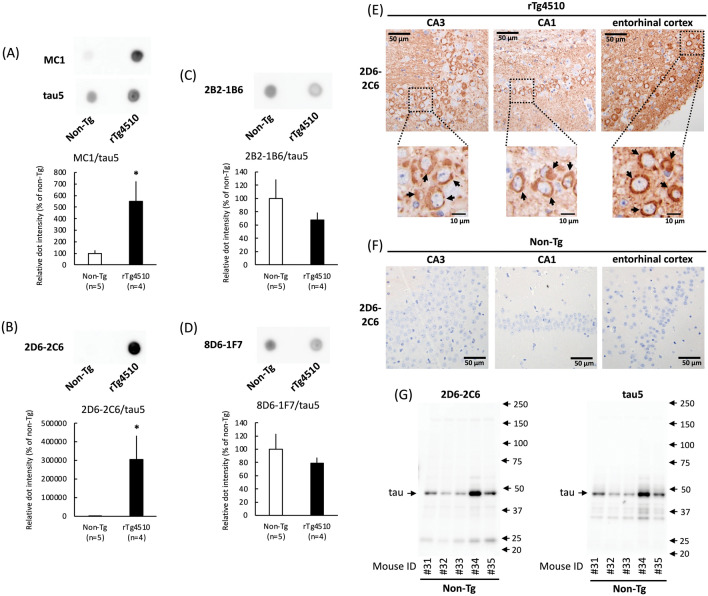
Binding of tau aggregation antibodies in an in vivo model. The TBS-soluble fraction was obtained from rTg4510 mice overexpressing human P301L mutant tau (0N4R) and from non-transgenic mice (non-Tg) at 10 months of age. This fraction was then subjected to dot blot (**A**–**D**), immunohistochemistry (**E**, **F**) and western blot (**G**) analyses. (**A**–**D**) The dot blot analysis detected tau in the occipital cortex homogenates from rTg4510 mice. The following antibodies were used: MC1 (0.74 μg/ml), which reacts with conformational tau aggregates (A, upper panel), pan-tau mouse monoclonal antibody (tau5; A, middle panel), and tau aggregation antibodies 2D6-2C6 (0.74 μg/ml) (**B**), 2B2-1B6 (**C**), and 8D6-1F7 (**D**). Densitometry of tau immunoreactivity was quantified. The levels of MC-1, 2D6-2C6, 2B2-1B6, and 8D6-1F7-reactive tau were normalized by the corresponding tau5-reactive tau levels. Quantitative data are presented as a percentage of non-Tg mice (mean ± SD of 4–5 mice). P values were determined using Student’s *t* tests. Significance is indicated by *(p < 0.05) shown on black columns comparing non-Tg with rTg4510 mice. (**E**, **F**) Immunohistochemistry using the 2D6-2C6 antibody highlighted accumulated tau aggregates (arrows) in the CA3 region (left panels), CA1 region (middle panels), and entorhinal cortex (right panels) of rTg4510 mice (**E**), but not of non-Tg mice (**F**). (**G**) In the western blot analysis, both 2D6-2C6 (left panel) and tau5 (right panel) detected endogenous tau in non-Tg mice.

Neurofibrillary tangle pathology increases with age in rTg4510 transgenic mice overexpressing human P301L mutant tau, a tau associated with frontotemporal dementia and parkinsonism linked to chromosome 17 (FTDP-17)^29^. The immunoreactivity of the three rat monoclonal antibodies with samples derived from the rTg4510 mice was assessed through dot blot analysis. The pan-tau antibody (tau5) binds both human and mouse tau^6^. In cell homogenates derived from both rTg4510 and non-Tg mice, the immunoreactivity intensity of tau5 in rTg4510 mice was higher than that in non-Tg mice (Fig. 2A). MC1 antibody can detect tau conformation changes along with tau aggregation in rTg4510 mice using dot blot analysis^30^ and immunohistochemistry^29^. The MC1 antibody, used as a positive control, showed 5.50-fold more immunoreactivity in rTg4510 mice than in non-Tg mice under normalization by tau5 (Fig. 2A). Surprisingly, 2D6-2C6 immunoreactivity was 3000-fold greater in rTg4510 mice than in non-Tg mice (Fig. 2B), indicating increased immunoreactivity to aggregated tau in vivo compared to that of MC1. Similar results were obtained from stepwise dilutions of samples taken from rTg4510 mice. The immunoreactivity of 2B2-1B6 (Fig. 2C) and 8D6-1F7 (Fig. 2D) were similar between rTg4510 and non-Tg mice. Therefore, we conducted further experiments using 2D6-2C6.

**Figure 3:**
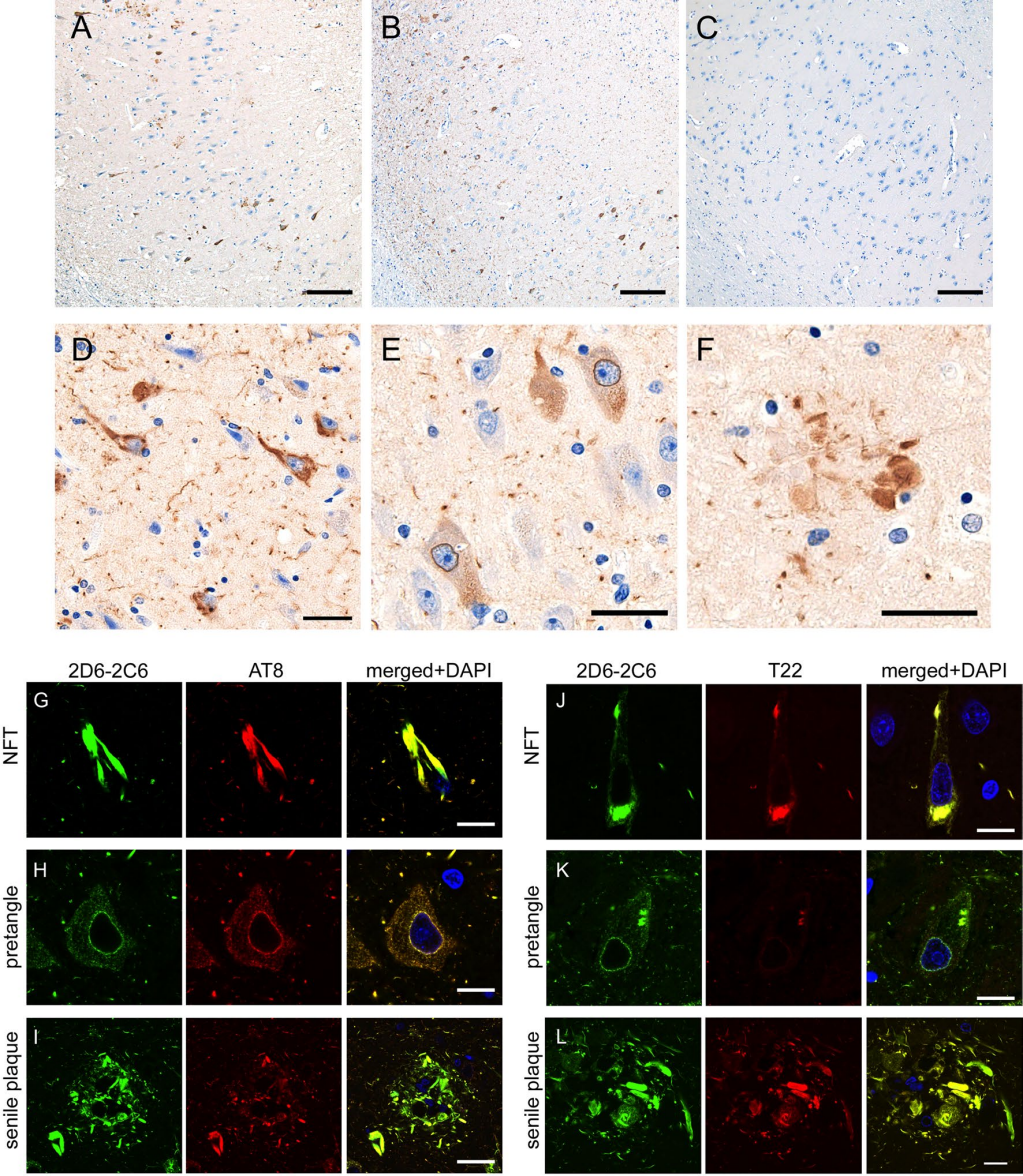
Immunohistochemistry and double immunofluorescence using 2D6-2C6 antibody on autopsied AD cases. (**A**–**F**) Immunohistochemistry using 2D6-2C6 antibody on autopsied AD cases. The panels (**A**–**C**) display CA1 of autopsied samples. Two cases with AD (**A**, **B**) demonstrated 2D6-2C6-immunopositivity in the CA1, whereas a neurologically healthy control (**C**) did not show dense aggregates. The 2D6-2C6 antibody immunolabeled NFTs (**D**), pretangles (**E**), and neuritic plaque (**F**) in the CA1. Black scale bars = 100 μm (**A**–**C**) and 20 μm (**D**–**F**). (**G**–**L**) Double immunofluorescence using 2D6-2C6 antibody combined with other anti-tau antibodies. All panels here are taken at the CA1 from the same AD case. Panels (**G**–**I**) indicate a combination of 2D6-2C6 and AT8, whereas panels (**J**–**L**) indicate a combination of 2D6-2C6 and T22. The 2D6-2C6 fluorescent signals were mostly merged with AT8 signals for NFT (**G**), pretangle (**H**), and neuritic plaque (**I**). The 2D6-2C6 and T22 antibodies also exhibited colocalization (**J**–**L**). Pretangle (**K**) was more sensitively detected by 2D6-2C6 antibody compared to T22. White scale bars = 10 μm.

The AT8 antibody, which recognizes phosphorylated paired helical filament tau at Ser202, Thr205, and Ser208^31^, reacted with tau aggregates in neuronal somata and neurites in hippocampal pyramidal cells of rTg4510 mice, but not in non-Tg mice (Supplemental Fig. 3)^29^. As with the AT8 antibody, immunostaining with the 2D6-2C6 antibody (Fig. 2E) was clear in regions containing CA3, CA1, and the entorhinal cortex, where tau aggregation was previously reported in rTg4510 mice^29^. This immunoreactivity was not observed in non-Tg mice (Fig. 2F). These results suggest that 2D6-2C6 binds tau aggregates in both immunohistochemical and biochemical analyses. To evaluate the immunoreactivity of 2D6-2C6 with mouse tau, non-Tg cerebral cortex samples were subjected to western blotting. Mouse tau was detected by both tau5 (Fig. 2G, right panel) and 2D6-2C6 (Fig. 2G, left panel), indicating that the binding of 2D6-2C6 is not human-specific. To confirm whether 2D6-2C6 recognizes tau aggregates in human subjects, we investigated the immunoreactivity of 2D6-2C6 using paraffin sections from two AD cases with Braak’s NFT stage V and a healthy control (Supplemental Table 1). 2D6-2C6-immunopositivity was densely observed in the hippocampus (Fig. 3A, B) and parahippocampal gyrus of two cases with AD but sparse in those of neurologically healthy control (Fig. 3C) by DAB staining. Expanded images showed that 2D6-2C6 immunolabeled flame-like NFTs (Fig. 3D), pretangles (Fig. 3E) and neuritic plaque (Fig. 3F); it is well known that neuritic plaques often exhibit tau epitopes as well as amyloid-β^32,33^. Double immunofluorescence revealed that the immunoreactivity of 2D6-2C6 overlaid that of AT8 (Figs. 3G–I, right panel) and T22 (Figs. 3J–L, right panel), which are anti-hyperphosphorylated tau and anti-oligomer tau antibodies, respectively. These results indicate that 2D6-2C6 binds phosphorylated tau aggregates including pretangles and NFTs in AD subjects.

Taken together, 2D6-2C6 is identified as a novel tau monoclonal antibody that is immunoreactive for tau aggregates including granular tau oligomers, with greater sensitivity than the MC1 antibody, and effectively stained human pretangles and NFTs.

## Epitope mapping of 2D6-2C6 on tau

**Figure 4:**
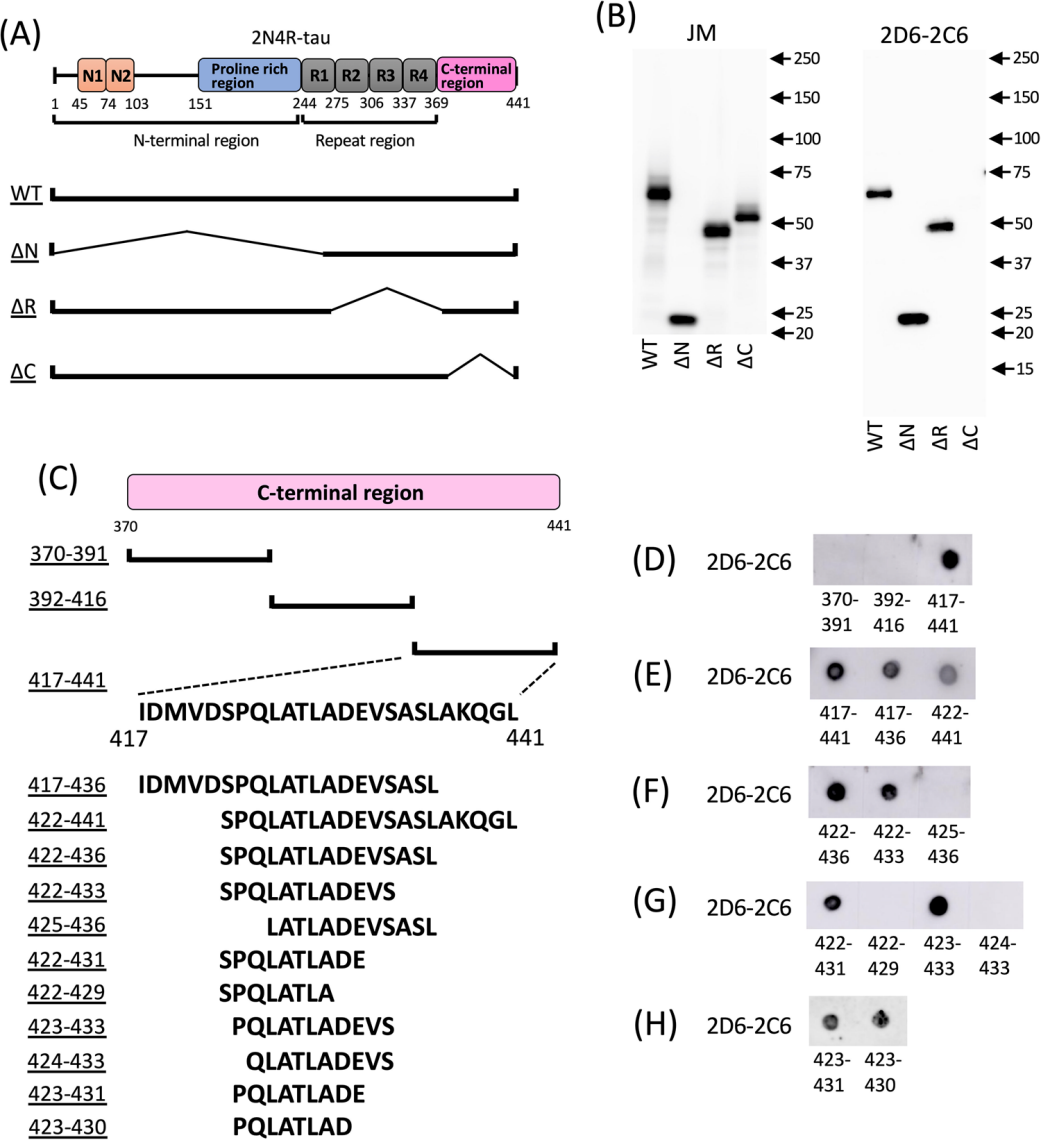
Epitope mapping of 2D6-2C6 on tau. (**A**) Schematic representation of human wild-type (WT) tau protein and its deletion mutants. The full-length 2N4R tau consists of an N-terminal region that includes a proline-rich region and N-terminal inserts, a microtubule binding repeat region, and a C-terminal region. The ΔN mutant lacks residues 1–243, the ΔR mutant lacks residues 244–369, and the ΔC mutant lacks residues 370–441. (**B**) cDNAs encoding WT, ΔN, ΔR and ΔC were transfected into cultured COS-7 cells. Tau proteins in the cell lysate were detected with the pan-tau antibody JM (B, left panel) and 2D6-2C6 (B, right panel) using SDS‒PAGE western blot analysis. (**C**) Schematic representation of peptides with partial sequences of the C-terminal region. (**D**–**H**) The peptides were dotted onto a nitrocellulose membrane and then reacted with 2D6-2C6.

To determine the 2D6-2C6 epitope of tau, we examined the binding of tau deletion mutants (ΔR, ΔN, and ΔC) (Fig. 4A) to 2D6-2C6 using SDS‒PAGE western blotting. The pan-tau antibody JM reacted with WT-tau and all three deletion mutants (Fig. 4B, left panel). However, 2D6-2C6 bound WT-tau, ΔN-tau, and ΔR-tau, but not ΔC-tau (Fig. 4B, right panel). This result indicates that the epitope of 2D6-2C6 is located in the C-terminal region (370–441 AAs) of tau. We further divided the C-terminal region into three segments (370–391, 392–416, and 417–441 AAs) and synthesized peptides corresponding to these sequences (Fig. 4C). Dot blot analysis showed that 2D6-2C6 binds to the peptide with AAs 417–441, but not to those with AAs 370–391 or 392–416 (Fig. 4D). Western blotting is not effective in detecting these peptides due to their short lengths. Additional peptides were synthesized by removing AA residues at the N- and/or C-terminal sides of the 417–441 sequence (Fig. 4C) and were subjected to dot blot analysis (Fig. 4E–H). The peptides with AAs 417–436 and 422–441 reacted with 2D6-2C6 (Fig. 4E), indicating that the epitope of 2D6-2C6 is located within the 422–436 AA sequence. Focusing on the C-terminal side of the 422–436 AAs, 2D6-2C6 bound to peptides with AAs 422–436 (Fig. 4F), 422–433 (Fig. 4F) and 422–431 (Fig. 4G), but not to peptides with AAs 422–429 (Fig. 4G), indicating that the epitope of 2D6-2C6 is within the 422–431 AA residues of tau. Focusing on the N-terminal sides, while 2D6-2C6 binding was observed in incubation with peptides with AAs 423–433 (Fig. 4G), 423–431 (Fig. 4H) and 423–430 (Fig. 4H), the signal was completely abolished by incubation with AAs 424–433 (Fig. 4G) and 425–436 (Fig. 4F). These results demonstrate that the epitope of the novel tau aggregation antibody 2D6-2C6 is the 423–430 AA sequence on tau.

### Binding of other tau antibodies against C-terminal regions with tau aggregates

**Figure 5:**
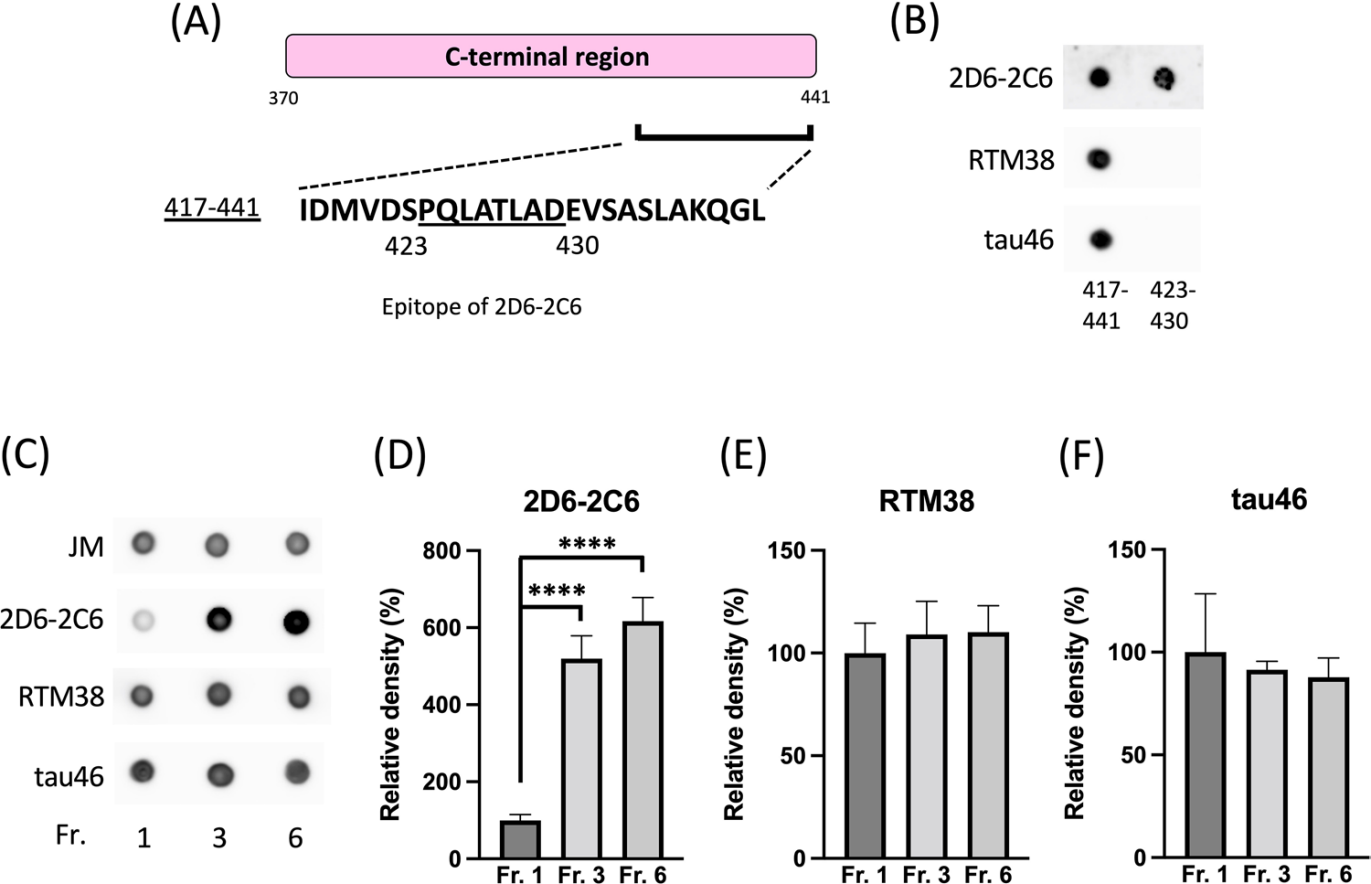
Binding of other antibodies against C-terminal regions with tau aggregates. (**A**) The sequence at amino acid residues 417–441 in the tau C-terminal region is illustrated. The epitope of 2D6-2C6 covers residues 423–430. (**B**) Peptides (417–441 and 423–430) were dotted onto a nitrocellulose membrane and probed with the 2D6-2C6 antibody, as well as other C-terminal monoclonal antibodies: RTM38 (immunogen: 417–441) and tau46 (epitope: 404–441). (**C**–**F**) Recombinant human full-length tau was polymerized using heparin and then fractionated. Monomer and multimer tau, granule tau oligomers, and longer fibrils are observed in Fraction 1, 3, and 6, respectively. A dot blot analysis detected tau in all three fractions using the pan-tau rabbit polyclonal antibody (JM), 2D6-2C6, RTM38, and tau46 (**C**). Tau immunoreactivity was quantified by densitometry (using ImageJ software). Levels of 2D6-2C6 (**D**), RTM38 (**E**), and tau46 (**F**)-reactive tau were normalized by the corresponding JM-reactive tau levels. Quantitative data are presented as a percentage of Fraction 1 (mean ± SD of 4 experiments). P values were determined using one-way ANOVA followed by Tukey’s multiple comparisons test. **** indicates p < 0.0001.

We evaluated the binding of antibodies to C-terminal regions with tau aggregates. Monoclonal tau antibodies, RTM38 (immunogen: 417–441)^34^ and tau46 antibody (epitope: 404–441)^35^ bound to the 417–441 peptide, but not to the 423–430 peptide (Fig. 5B), indicating that the epitopes of RTM38 and tau46 are either the 417–422 or 431–441 AA sequences. To evaluate the binding of RTM38 and tau46 with tau aggregates, we prepared recombinant monomeric tau (Fraction 1), granular tau oligomers (Fraction 3) and fibrils (Fraction 6) and conducted dot blot assays (Fig. 5C). Unlike 2D6-2C6 (second image from top of Fig. 5C, D), both RTM38 (second image from bottom of Fig. 5C, E) and tau46 (lower image of Fig. 5C, F) bound equally with tau in Fraction 1, Fraction 3 and Fraction 6. These data indicate that RTM38 and tau46, which are anti-tau antibodies against the C-terminal region with epitopes outside the 423–430 AA residues, serve as pan-tau antibodies but not as tau aggregation antibodies. Taken together, the 423–430 AA residues in the C-terminal regions are particularly exposed upon aggregation and are specifically recognized by the tau aggregation antibody 2D6-2C6.

## Discussion

Elucidating the formation of granular tau oligomers is essential for a better understanding of tauopathies^19,21,23,36^. In this study, we developed a novel monoclonal antibody, 2D6-2C6, that binds tau aggregates specifically, including granular tau oligomers, but not normal tau. Furthermore, 2D6-2C6 detected tau aggregates in the brains of AD subjects and in an in vivo mouse model. The epitope of 2D6-2C6 is located at AAs 423–430 in the C-terminus region of tau. While several antibodies targeting tau oligomers have been reported previously, this study is the first to identify an antibody that recognizes the C-terminal region of tau and binds tau aggregates specifically.

### Novelty of 2D6-2C6 compared to existing tau aggregation antibodies and other well-characterized antibodies

The A11 polyclonal antibody recognizes the common conformation of amyloidogenic protein oligomerization independent of AA sequence^37^. In dot blot analysis, A11 bound recombinant tau fibrils but not oligomers, including granular tau oligomers^19^. A 2B10 monoclonal antibody was generated against low-n tau oligomers using the mutant tau repeat region with the deletion of lysine 280 (TauRDΔK). The antibody penetrated cells and inhibited intercellular tau oligomerization^38^. Although its epitope was not identified, the antibody likely binds the microtubule-binding region on tau. T22 polyclonal antibody and TORC1 monoclonal antibody were identified by Kayed and colleagues as tau oligomer antibodies^39,40^. These antibodies detect oligomeric tau by SDS‒PAGE western blot analysis, which suggests that the conformation of the antibody-positive tau oligomers are resistant to high concentration detergents. However, no epitopes have been reported for either antibody^39,40^. Pretangle appears to be more sensitively detected by 2D6-2C6 antibody compared to T22. TNT2 binds to the phosphatase-activating domain (7–12 AA sequence) on tau and detects early pretangle tau pathology in AD human subjects. Because TNT2 does not bind late-stage tangles, the epitope of TNT2 is lost with tangle evolution^41^. Tau phosphorylation antibodies such as AT8 and PHF-1 are well-known antibodies that react with pathological tau. Recent studies have reported that AT8 is more selective for pathological tau than PHF-1^42^. AT8 reported to exhibit nonselective binding in mouse brain samples^43^. 2D6-2C6 is likely an antibody that detects conformational changes independent of phosphorylation. MC1 and TOC1 are known tau conformation antibodies. MC1, a mouse monoclonal antibody, recognizes the pathological conformation of tau in vitro, in vivo, and in AD human subjects^44,45^. In vitro experiments showed that MC1 is immunoreactive to tau oligomers to tau fibrils (Supplemental Fig. 4)^19^, with its epitope located in discontinuous tau AA sequences in both the N-terminus (7–9 AAs) and R3 (313–322 AAs)^45^. In vitro analyses reveal that 2D6-2C6, similar to MC1 (Supplemental fig. 4)^19^, is capable of detecting both tau fibrils and granular tau oligomers. Conversely, in vivo, the binding affinity of 2D6-2C6 for tau aggregates surpasses that of MC1, suggesting that 2D6-2C6 is highly effective in detecting tau aggregation compared to MC1. TOC1 is a mouse monoclonal antibody whose immunogen was a crosslinked dimer tau^46^. Its epitope is located between the 209–224 AA sequence on tau^47^. While TOC1 binds oligomeric tau, no reports demonstrate that TOC1 binds to tau fibrils. 2D6-2C6 is an antibody that captures fibers from granular tau oligomers rather than dimers, suggesting it may detect periods closer to disease progression. Tau5 and tau46 are general-purpose tau antibodies, but their reactivity was recently reported to decrease with tau phosphorylation^42^. In contrast, 2D6-2C6 antibodies have a better signal-to-noise (S/N) ratio in vivo than in vitro, speculating that, unlike these general-purpose antibodies, 2D6-2C6 reactivity is not decreased by phosphorylation. Antibodies targeting tau aggregation that specifically recognize C-terminal regions have not been reported. In contrast to the previous tau aggregation antibodies, our novel discovery, 2D6-2C6, is an antibody that is distinctively immunoreactive to the C-terminal region of tau. A recent study established a resource designed to guide informed antibody choice for tau detection^42^. To detect total tau, an optimal strategy might employ a combined panel of N-terminal, mid-domain and C-terminal antibodies. To our knowledge, there are no tau aggregation antibodies that recognize the C-terminus. This may have hindered optimal research strategies for studying the mechanism of tau aggregation. The 2D6-2C6 antibody found in this study can help advance this area of research.

### Tau aggregation and the C-terminal region of tau

Several reports have shown a role of the C-terminal region of tau in aggregation. Tau phosphorylation at S422^48,49^ and S396^50^ in the C-terminal region was observed in mild cognitive impairment and the early stages of AD. Furthermore, substitutions with negatively charged AAs to act as pseudophospho-mimetics S422E^51^, and S396E/S404E^52^ increases tau aggregation in vitro. The C-terminus region containing Tau-CTF24 (residues L243-L441) cleaved by calpain accelerated heparin-induced tau fibrillization ability compared to full-length tau^53^. Overexpression of ΔD421-tau cleaved by caspase also promoted oligomerization in the brains of mice^54^. While tauC3 antibody-positive Δ421 tau has been observed in the brains of AD patients^49,55^, tauC3 binds mature tangles rather than pretangles. This indicates that tau is cleaved after the formation of tau fibrillization^56^. Results of a recent report show that the C-terminus region is exposed outside the repeat region when 0N3R recombinant tau is aggregated^57^. These previous reports and our results suggest that exposure of the 423–430 AA sequence associated with tau aggregation occurs in early AD and MCI. Cryo-EM revealed structural cores in 3R + 4R tauopathies (AD, CTE) , 3R tauopathy (PiD) , and 4R tauopathies (PSP, CBD, GGT, GPT, AGD)^58–60^. In these tauopathies, the core was included in R1-R4 and extended to approximately 18 AA residues from the C-terminal region of tau. An experiment with AFM detected thick fibrils in full-length tau aggregates compared with tau consisting of only the repeat region^61^. These results indicate that, in tau aggregation, sequences other than the core structure constitute a structural conformation, which is recognized by 2D6-2C6. In human AD brain slices, 2D6-2C6 immunoreactivity co-localizes with that of AT8 (see Fig. 3), which detects phosphorylated tau aggregates, indicating that tau 423–430 AA residues recognized by 2D6-2C6 are externally exposed during tau aggregation in human samples.

Other monoclonal antibodies, such as RTM38^34^ and tau46^35^, which recognize the C-terminal sequences of tau, have been reported. Interestingly, these antibodies have a different epitope from 2D6-2C6 and bind equally to both non-aggregating and aggregating tau (see Fig. 5). These observations indicate that only the 423–430 AA sequence in the C-terminus of tau is exposed during aggregation. Furthermore, 2D6-2C6 provides valuable insights into one aspect of the tau aggregation mechanism. The reason why we could produce tau aggregation antibodies with a new mechanism might stem from our use of β-sheet-positive granular tau oligomers as the immunogen. This approach contrasts that of previous reports that used β-sheet-negative oligomers containing cross-linked tau dimers^46^ and low-n oligomers^38^ as immunogens.

2D6-2C6 binds more strongly with tau aggregates in vivo than in vitro. Unlike in vitro, in vivo tau undergoes posttranslational modifications, including phosphorylation and acetylation. The paper clip conformation of tau formed by pseudophosphorylation at the AT8, AT100, and PHF1 sites is bound by the MC1 antibody^62^. Tau phosphorylation at S422, which is located near the vicinity of the 2D6-2C6 epitope, enhances the formation of SDS-stable dimers^51^. These observations suggest that the negative charge from phosphorylation at S422 may facilitate exposure of the 423–430 AA sequence in tau.

### Granular tau oligomer antibody as a diagnostic tool for tauopathies

Tau antibodies are essential tools in biomarker analysis of AD. For example, tau phosphorylation at T217 and T181 in plasma and CSF is increased in AD and the preclinical stage of AD compared with controls^63,64^. Goedert and colleagues reported that tau aggregates with a ring-like structure from 0N4R P301S tau transgenic mice by sucrose gradient centrifugation increased tau seed assembly in cultured cells^27^. Furthermore, vesicle tau oligomers with globular particles from AD patients spread tau pathology in vitro and in vivo^18^. OptoTau, a tau protein fused with CRY2olig, is a light-sensitive protein that can form homo-oligomers that form granular tau oligomers in cells and act as a seed for tau fibrils in vitro^65^. Our previous report showed that granular tau oligomers were observed in Braak stage I AD^23^. Thus, the 2D6-2C6 antibody against granular tau oligomers may be useful for the detection of preclinical biomarkers for AD.

### Limitations of the study

2D6-2C6 is not a human-specific antibody because it also reacts with mouse tau. Even AT8, which can well detect tau pathology, has previously been reported to show nonselective binding in mouse brain samples. This implies that even if the antibody reacts with mouse tau, it does not completely negate the significance of the antibody in detecting human pathology.

In vitro, 2D6-2C6 is significantly more reactive to aggregated tau than to non-aggregated tau, though it is not completely insensitive to monomeric tau. However, the S/N ratio of 2D6-2C6 is better in vivo than in vitro. This result may be attributed to in vivo conditions, which include factors such as phosphorylation and interaction with other molecules, that are not present in vitro.

This study may not have sufficiently examined tau phosphorylation because 2D6-2C6 was produced by immunization using recombinant tau aggregates. However, in human AD subjects, 2D6-2C6 co-localized with neurons stained by the tau phosphorylation antibody AT8, which recognizes pretangles and NFTs, indicating that 2D6-2C6 reacts with phosphorylated tau aggregates. Tau phosphorylation at the T427 AA residue is strong in AD patients and FTLD-tau patients compared with healthy controls^66^. The T427 AA residue lies within the epitope of 2D6-2C6 (423–430 AA sequence). Thus, targeting the phosphorylation of the 2D6-2C6 epitope may be a diagnostic and therapeutic strategy for tauopathies by removing toxic tau oligomers but not normal tau.

In conclusion, our results show that the C-terminal 423–430 AAs of tau are positioned outward during the formation of granular tau oligomers. Thus, 2D6-2C6 antibodies targeting the 423–430 AA sequence may be promising for the early diagnosis of tauopathies.

## Methods

### Materials

Full-length human 2N4R tau cDNA was inserted into the pRK172 vector for recombinant tau preparation^19^. Wild-type 2N4R and mutant forms of 2N4R tau with deletions of the repeat region (ΔR) at 244–369 AAs, the N-terminal region (ΔN) at 1–243 AAs, and the C-terminal region (ΔC) at 370–441 AAs were inserted into the pCI-neo vector (Promega, Madison, Wisconsin, USA) for transient transfection (Fig. 4A). Peptides (> 50% purity) were obtained from Cosmo Bio Co., Ltd (Koto-ku, Tokyo, Japan). Chemical reagents were purchased from Nacalai Tesque Inc. (Kyoto, Japan), FUJIFILM Wako Pure Chemical Corp. (Osaka, Japan), and Sigma‒Aldrich (St. Louis, Missouri, USA).

### Preparation of recombinant tau protein

*Escherichia coli* (*E.coli*) BL21 (DE3) (BioDynamics laboratory Inc., Bunkyo-ku, Tokyo, Japan) transformed with the full length or mutant cDNA and cultured. Tau protein expression was induced by the addition of isopropyl-β-D-1-thiogalactopyranoside (Nacalai Tesque Inc.). To obtain a crude fraction, *E. coli* expressing tau were sonicated and boiled. The fraction was purified using Cellufine Phosphate (JNC Corp., Chiyoda-ku, Tokyo, Japan), ammonium sulfate fractionation, an NAP10 column (GE Healthcare, Chicago, USA), and reversed-phase-HPLC (COSMOSIL Protein-R Waters; Nacalai Tesque Inc.). After freeze-drying, recombinant tau protein was dissolved in Milli-Q water^19^. Tau protein concentration was determined with Coomassie brilliant blue (CBB) stain using bovine serum albumin (BSA) as a protein standard.

### Tau polymerization reaction

Recombinant 2N4R tau protein (10 μM) and thioflavin T (10 μM), which can detect β sheet-rich amyloid structures, were mixed in buffer containing HEPES (10 mM, pH = 7.4) and NaCl (100 mM). Tau polymerization was induced by the addition of heparin (0.06 mg/ml; Acros Organics, Antwerp, Belgium) and incubation at 37 ℃. Fluorescence resulting from the binding of thioflavin T with aggregated tau was monitored using a BioTek Synergy HTX multimode reader (excitation wavelength, 444 nm; emission wavelength, 485 nm) (Agilent, Santa Clara, California, USA). Samples were recovered at various times after tau polymerization reactions.

### Sucrose step gradient centrifugation

Sucrose density gradient centrifugation was conducted based on a previously described method^19^ with minor modifications. Briefly, a sucrose gradient solution consisting of four layers (20, 30, 40 and 50% sucrose) was prepared in buffer containing HEPES (10 mM, pH = 7.4) and NaCl (100 mM). The polymerized tau was layered on top of the sucrose gradient solution. Following centrifugation (50,000 rpm, for 2 h using either an MLS50 rotor (Beckman Coulter, Brea, California, USA) or a TLS55 rotor (Beckman Coulter). Each layer was collected as Fractions 1 through 5, while the pellet was recovered as Fraction 6.

### Preparation of rat monoclonal antibodies against granular tau oligomers

For buffer exchange, Fraction 3 was subjected to centrifugation at 50,000 rpm using TLA55 (Beckman Coulter) for 1 h. Subsequently, the pellets containing granular tau oligomers were resuspended in PBS. The tau protein concentration in the resuspension was determined using CBB staining. A rat was immunized with an initial dose of 125 µg of the granular tau oligomers, followed by a booster injection of 87 µg 16 days later. Four days post booster, the rat was sacrificed. Utilizing the iliac lymph node method^24,25^, rat monoclonal antibodies were produced. Initial screening of the antibodies derived from hybridoma colonies for binding to granular tau oligomers was performed using ELISA.

### Atomic force microscopy (AFM)

AFM can observe the morphology of recombinant tau aggregates^19^. After completing the tau polymerization reaction, the recovered tau aggregates were diluted to a concentration of 1 μM in Milli-Q water. The samples were then deposited onto mica sheets and incubated for 10 min in a moisture box. After rinsing with Milli-Q water, the tau aggregates were visualized using a cantilever (either OMCL-TR400PSA or BL-AC40TS-C2; Olympus, Tokyo, Japan) on an SPM 9700 instrument (Shimadzu, Kyoto, Japan) under dynamic mode.

### Animals

rTg4510 mouse^6^, a transgenic model for human tauopathy, expresses human tau 0N4R isoform containing the frontotemporal dementia associated P301L mutation. The rTg4510 mice were generated by crossing the responder line (#015815, Jackson Laboratory, Bar Harbor, Maine, USA) with an activator line (#016198, Jackson Laboratory). The responder line, carrying a tau^P301L^ cDNA downstream of a tetracycline operon–responsive element (TRE), was maintained on the FVB/N background. The activator line, expressing a tetracycline-controlled transactivator (tTA) under the control of the CaMKIIα promoter, was maintained on the C57BL/6 J background. The rTg4510 and non-transgenic mice (FVB/N-C57BL/6 J), male and female, were individually housed and received food ad libitum. The mice were kept under standard conditions with a constant temperature of approximately 25 ℃ and a 12 h:12 h light/dark cycle. At 10 months of age, the mice (rTg4510 mice, n = 4; non-transgenic mice, n = 5) were anesthetized with isoflurane and sacrificed. The left hemisphere was placed in 10% formalin, from which paraffin blocks were produced. The occipital cortex was extracted from the right hemisphere for biochemical assays and stored at -80 ℃.

### Preparation of a TBS-soluble fraction from mouse brain

The occipital cortex from rTg4510 mice was homogenized in cold TBS buffer containing protease inhibitors and phosphatase inhibitors. After homogenization, the mixture was centrifuged (23,000 rpm, 15 min, 4 ℃) using a TLA55 rotor (Beckman Coulter). The supernatant was stored at −80 ℃ until use in biochemical experiments.

### Dot blot analysis

Dot blot analysis was employed to detect tau in the samples. These samples included Fractions 1–6 from the sucrose gradient step centrifugation and the TBS-soluble fraction from the occipital cortex of mice. Samples (1–2 µL) were loaded onto nitrocellulose membranes (Cytiva, Tokyo, Japan). The membranes were then blocked with 5% milk at room temperature for 1 h, followed by incubation with antibodies against granular tau oligomers, as well as pan-tau antibodies such as JM (immunogen, 2N4R-tau;^28^), tau5 (epitope, tau 210–241 AA residues; Invitrogen, AHB0042, Waltham, Massachusetts, USA ), RTM38 (immunogen, 417–441 AA residues; FUJIFILM Wako Pure Chemical Corp., 017–26893) and Tau46 (epitope, 404–441 AA residues; Invitrogen, 13–6400), for overnight at 4 ℃. Secondary antibodies (Jackson ImmunoResearch, West Grove, Pennsylvania, USA) conjugated with horseradish peroxidase were added to the membranes and incubated at room temperature for 2 h. Chemiluminescence was detected using the Chemi-Lumi One series (Nacalai Tesque, Kyoto, Japan) on an Amersham Imager 600 (Cytiva, Marlborough, Massachusetts, USA). Dot intensity was quantified by defining regions of interest (ROIs) in the image as circles and measuring the mean gray value using ImageJ. Raw data are shown in Supplemental Fig. 5.

### Western blot

TBS soluble fractions from mice were suspended in Laemmli sample buffer containing 2-mercaptoethanol and boiled for 10 min. The samples were loaded and separated on a SuperSep Ace 5–20% gel (FUJIFILM Wako Pure Chemical) and subsequently transferred to nitrocellulose membranes (Cytiva). Next, proteins were detected using a procedure similar to the dot blot analysis. Raw data are shown in Supplemental Fig. 5.

### Human samples and analysis

We included two autopsied cases with AD (two males who died at the age of 84 and 87, respectively) and a control without any neurological disorders (a male who died of pneumonia at the age of 67). The demography of included subjects was summarized in Supplemental Table 1. Autopsies were undertaken after obtaining of written informed consent from family members in accordance with ethical committee of Aichi Medical University. The brains were fixed in 20% formalin for at least a month, followed by standardized trimming and paraffin embedding. Sections of hippocampus at the lateral geniculate nucleus underwent immunohistochemical analyses. The 2D6-2C6 anti-tau oligomer antibody was used with 1:100 dilution overnight, and antigen retrieval was taken with 98℃ citrate buffer (pH6.0) for 25 min. We undertook secondary immunolabeling using a standard avidin-biotin method.

We also performed double immunofluorescence using 2D6-2C6 antibody coupled with an anti-hyperphosphorylated tau mouse monoclonal antibody (AT8, 1:500, Thermo Fisher, Waltham, MA) or an anti-tau-oligomer rabbit polyclonal antibody (T22, 1:500, Millipore, Burlington, MA). Anti-rat, mouse, or rabbit IgG coupled with Alexa 488 or 568 was used for secondary immunolabeling. Immunofluorescent specimens were observed using a laser-confocal microscope (FV-3000, Olympus, Tokyo Japan) under the same settings for each coupling of primary antibodies.

### Immunohistochemistry for mouse samples

Tau in paraffin sections from the brains of mice was detected using the AT8 antibody (phospho-Ser202, Thr205, Ser208) and the 2D6-2C6 antibody, following the protocol described previously^67^.

### Statistical analysis

The results are expressed as the means ± SDs. The significance of differences between two groups was evaluated using Student’s or Welch’s t tests. For comparisons involving three or more groups, one-way analysis of variance followed by Tukey’s multiple comparisons test was employed. All analyses were performed using PRISM9 (GraphPad Software Inc., Boston, Massachusetts, USA). p < 0.05 was considered statistically significant.

### Ethical statements

All animal experiments and procedures were reviewed and approved by the Gakushuin University Animal Experimentation Committee (Permit No. 17). All animal experiments were performed in accordance with Japanese and university guidelines and regulations for the care and use of experimental vertebrate animals. The authors have complied with the ARRIVE guidelines 2.0 for reporting. All recombinant DNA experiments were reviewed and approved by the Gakushuin University Recombinant DNA Experiment Safety Committee (Permit No. R4-12). Human experiment was performed in line with the regulations outlined in the Declaration of Helsinki (WMA, 2013), and the research-purpose archiving of autopsied subjects was approved by the Ethical Committee of Aichi Medical University (AKBRC 2019-M019).

### Supplementary Information

Supplementary Information.

## Data Availability

The datasets used and/or analysed during this study available from the corresponding author on reasonable request.
