# Supplementary material for: A novel monoclonal antibody generated by immunization with granular tau oligomers binds to tau aggregates at 423-430 amino acid sequence
Source: Sci Rep. 2024 Jul 26;14:16391. doi: 10.1038/s41598-024-65949-7 (PMC11282240; doi:10.1038/s41598-024-65949-7)
Supplement: Supplementary file 1 — Supplementary Information. [file 41598_2024_65949_MOESM1_ESM.pdf]

**A Novel Monoclonal Antibody Generated by Immunization with Granular Tau Oligomers Binds to Tau Aggregates at 423-430 Amino Acid Sequence**

Yoshiyuki Soeda, Emi Hayashi, Naoko Nakatani, Shinsuke Ishigaki, Yuta Takaichi, Taro Tachibana, Yuichi Riku, James K. Chambers, Riki Koike, Moniruzzaman Mohammad, Akihiko Takashima.

**Supplemental information**

**Supplemental figure 1**

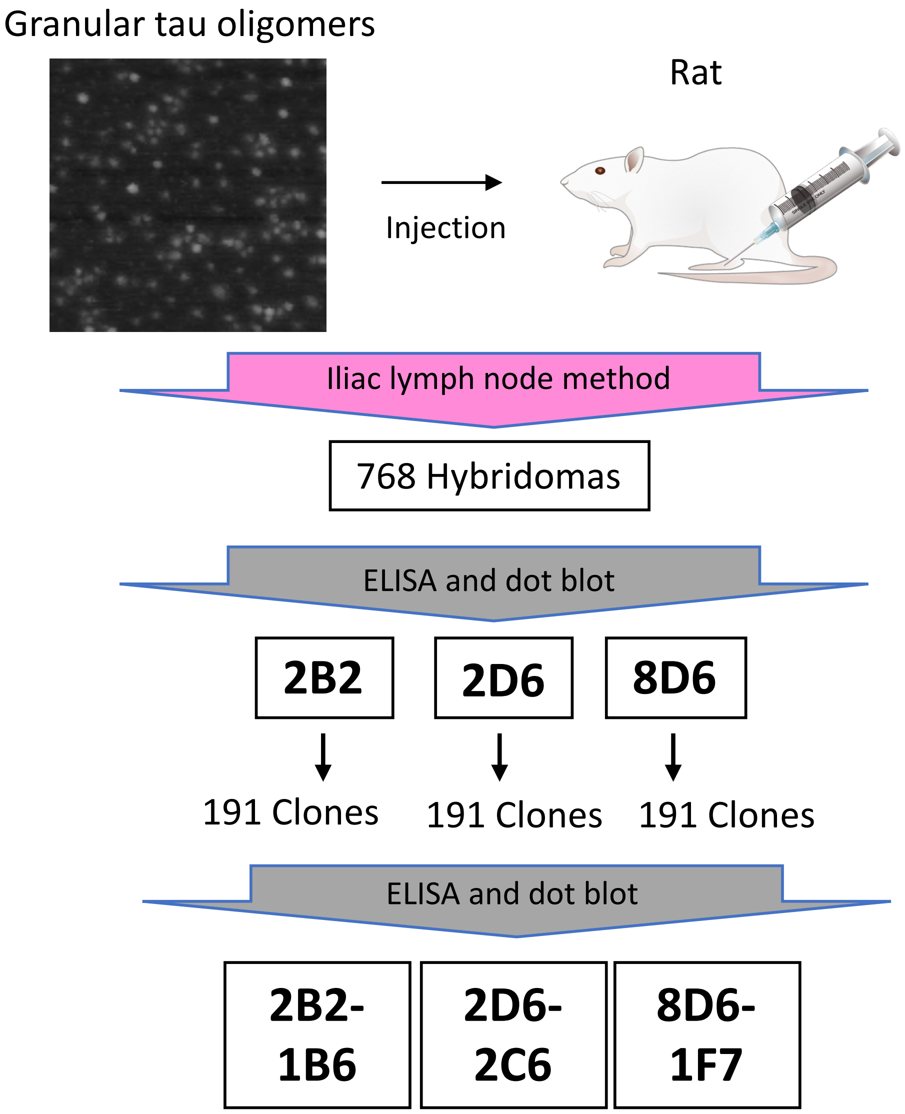


**Supplemental figure 1: Screening of antibodies against granular tau oligomers.** A rat was immunized by injection with granular tau oligomers. Using the iliac lymph node method, 768 hybridomas were produced. In ELISA and dot blot assays, the medium from three hybridomas (2B2, 2D6, 8D6) showed strong binding to granular tau oligomers. From these, 191 clones were derived by culturing a single cell from each hybridoma. Upon repeating the ELISA and dot blot assays, rat monoclonal antibodies from hybridoma colonies 2B2-1B6, 2D6-2C6, and 8D6-1F7 were identified as specific antibodies targeting granular tau oligomers. Images of the rat and the injection needle were provided free of charge by the National Bioscience Database Center (© 2016 DBCLS TogoTV/CC-BY-4.0).

## Supplemental figure 2

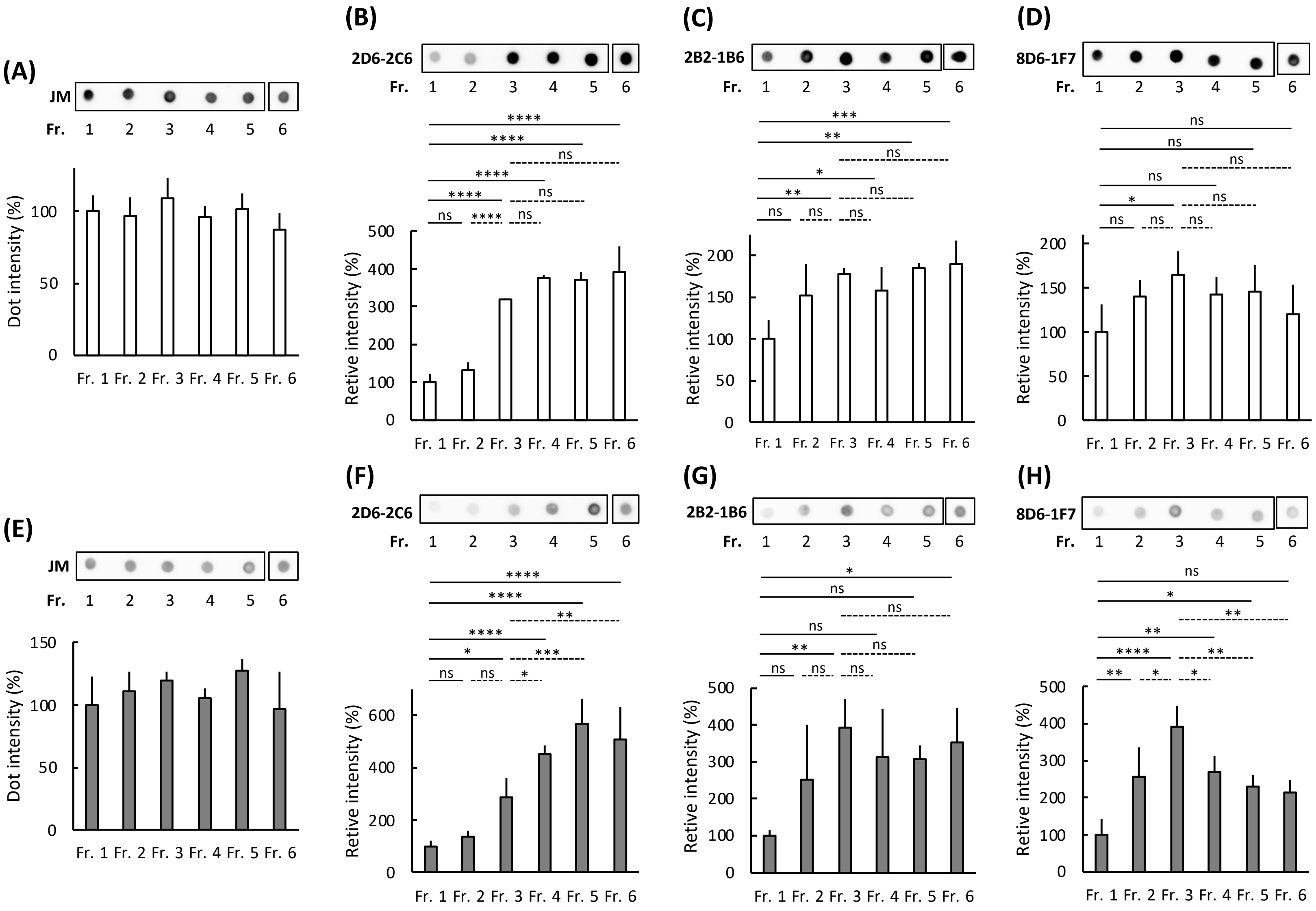

**Supplemental figure 2: Binding of antibodies to fractionated recombinant tau aggregates at various concentrations.**

Recombinant human full-length tau was polymerized and then fractionated into fractions (Fr) 1-6 using sucrose step gradient centrifugation. Dot blot assays detected tau presence in Fr 1-6 using a pan-tau rabbit polyclonal antibody, JM (A and E), and monoclonal antibodies 2D6-2C6 (B, F), 2B2-1B6 (C, G), and 8D6-1F7 (D,H) derived from rat immunized with granular tau oligomers. Fraction 1 contained tau concentrations of 200 μg/ml (A-D) and 50 μg/ml (E-H). Tau immunoreactivity was quantified using densitometry. The levels of tau reactivity for 2D6-2C6, 2B2-1B6, and 8D6-1F7 were normalized to the corresponding JM-reactive tau. Quantitative data are presented as a percentage of Fr. 1 (mean ± SD from four experiments). P values were determined using one-way ANOVA followed by Tukey's multiple comparisons test. Significance levels are indicated as * ($P<0.05$), ** ($p<0.01$), *** ($p<0.001$), and **** ($p<0.0001$). "ns" denotes not significant. Significance is indicated on solid lines for comparisons of Fr. 1 versus Fr. 2, 3, 4, 5, or 6 and on dotted lines for Fr. 3 versus Fr. 2, 4, 5, or 6. The dotted signals of Fr. 6 were cropped from different parts of the same membrane (Supplemental Figs. 5A-D).

## Supplemental figure 3

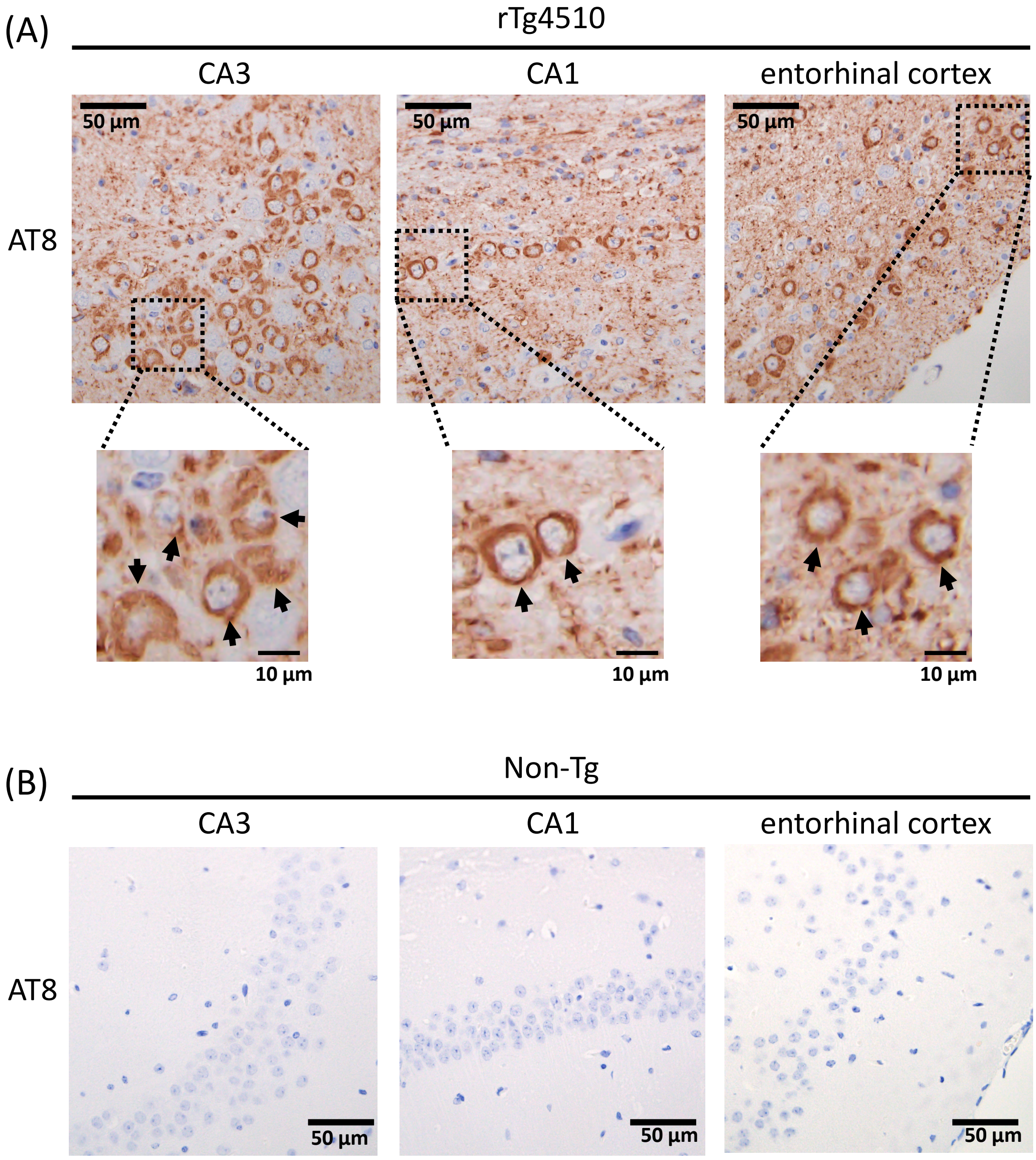


**Supplemental Figure 3: Immunostaining of tau pathology in rTg4510 mice using the phospho-Tau antibody AT8.** Paraffin sections from rTg4510 mice and from non-transgenic mice (non-Tg) at 10 months of age were immunostained with the AT8 antibody. This antibody specifically recognizes phosphorylated paired helical filament tau at Ser202, 205, and 208 [1]. (A) Accumulated tau aggregates (arrows) were observed in the cell bodies of the CA3 (left side of the panels), CA1 (center of the panels) and entorhinal cortex (right side of the panels) regions from rTg4510 mice. (B) No such aggregates were observed in the non-transgenic mice.

**Supplemental figure 4**

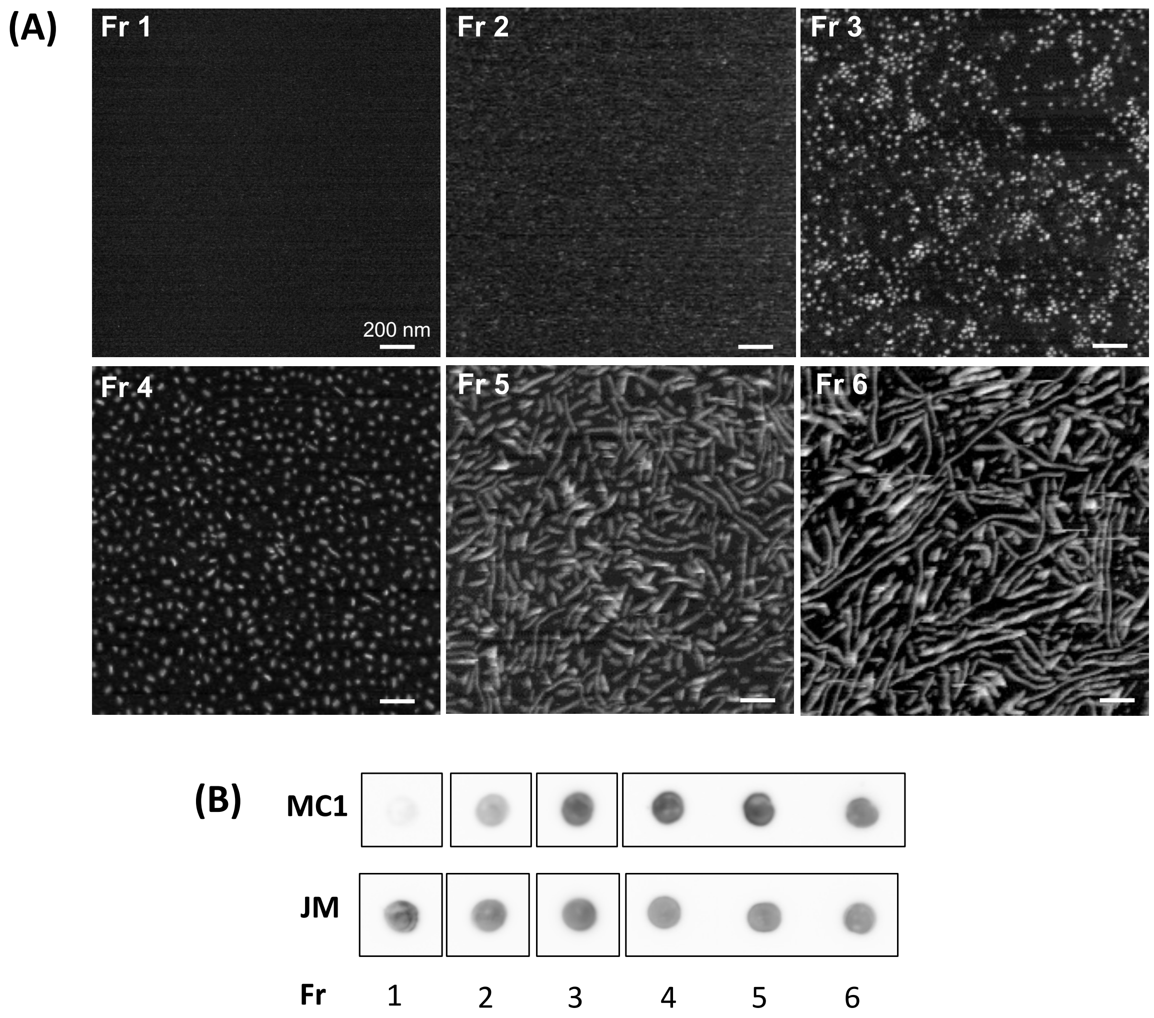

**Supplemental Figure 4: MC1 antibody binding to fractionated recombinant tau aggregates.** Full-length recombinant human tau polymerized by heparin was fractionated into fractions (Fr) 1-6 by sucrose step gradient centrifugation. Fr 1, Fr 3, and Fr 4-6 contain monomeric and multimeric tau, granular tau oligomers, and fibrils, respectively [2]. (A) Tau morphology was observed with Atomic Force Microscopy (AFM). White scale bars = 200 nm. (B) Tau was detected by dot blot analysis using the tau conformation antibody MC1 (upper panel) and the pan-tau antibody JM (lower panel). These results replicate those reported by Maeda and his colleagues [2], confirming that the size fractionation of the tau multimer is working properly in this study. The dotted signals of Fr. 1, 2 and 3 were cropped from different parts of the same membrane (Supplemental Figs. 5E and F).

# Supplemental figure 5

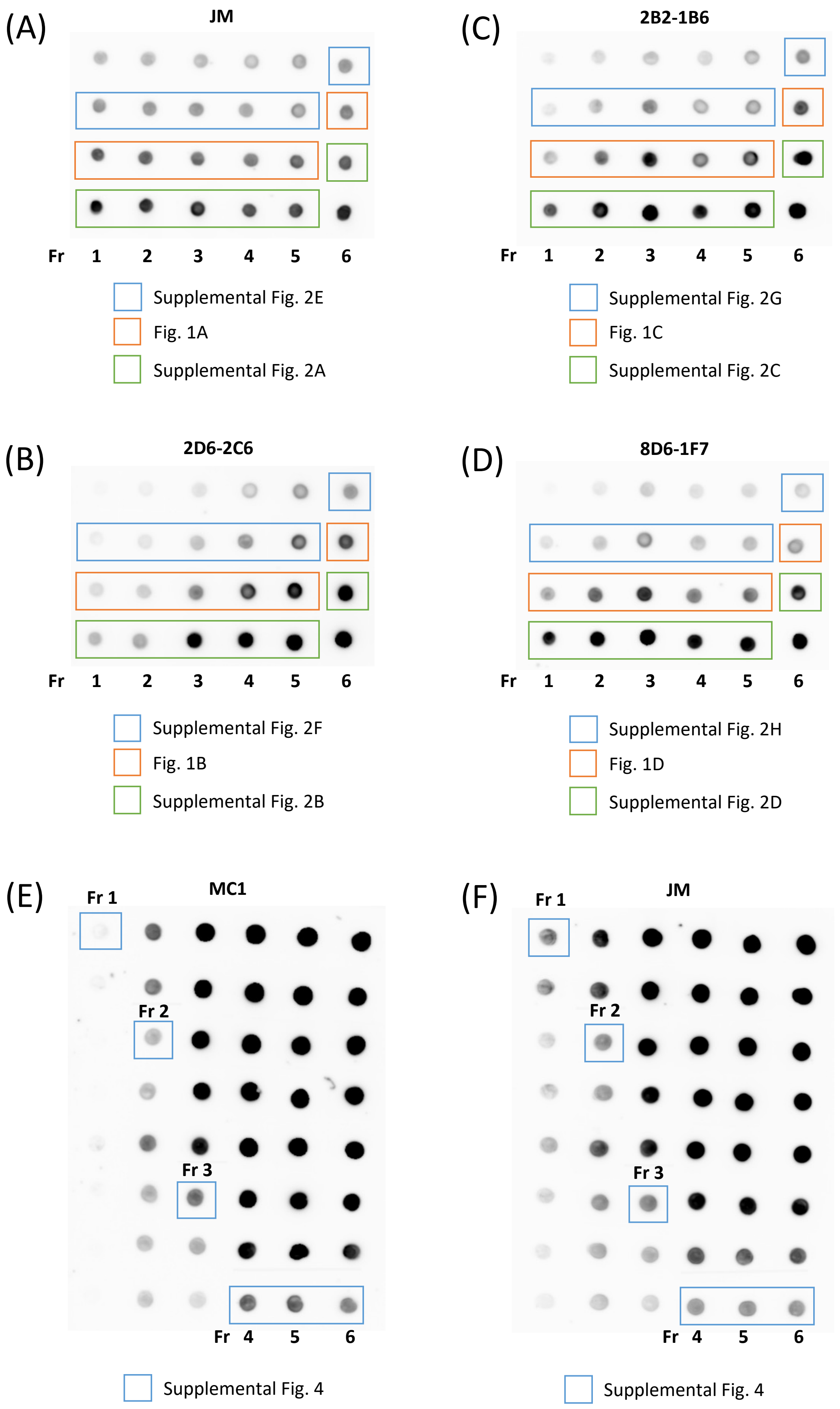

**Supplemental figure 5 continued**

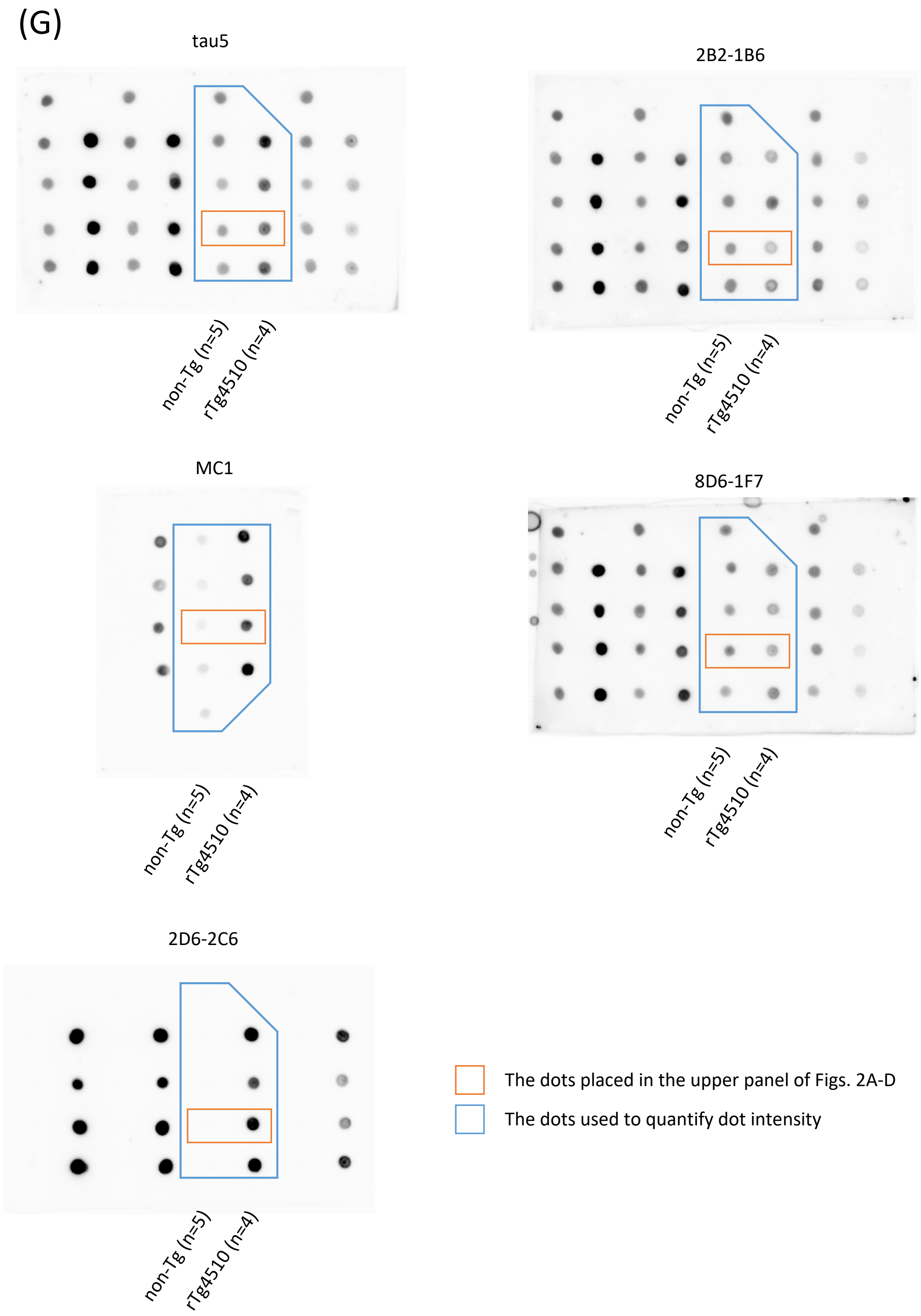

**Supplemental figure 5 continued**

(H)

2D6-2C6

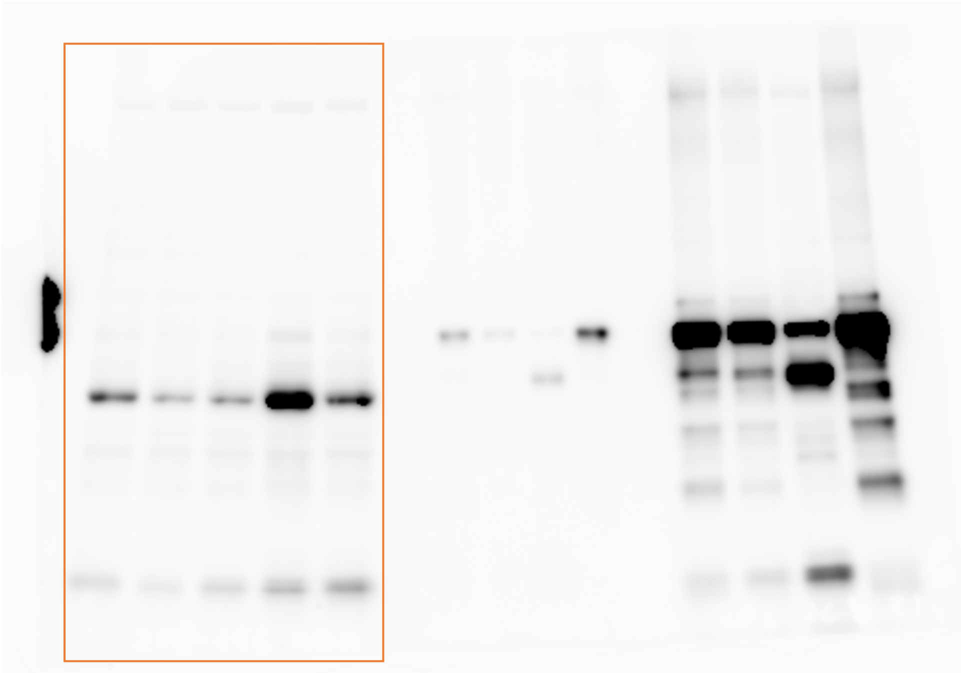

Tau5

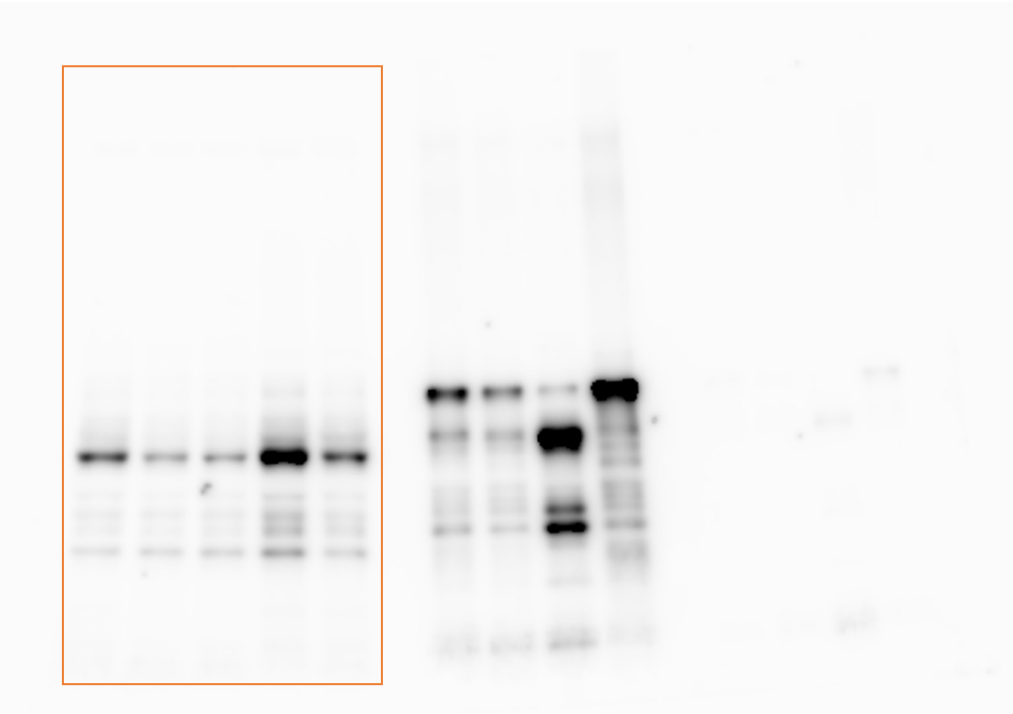

The bands placed in the Fig. 2G

**Supplemental figure 5 continued**

(I)

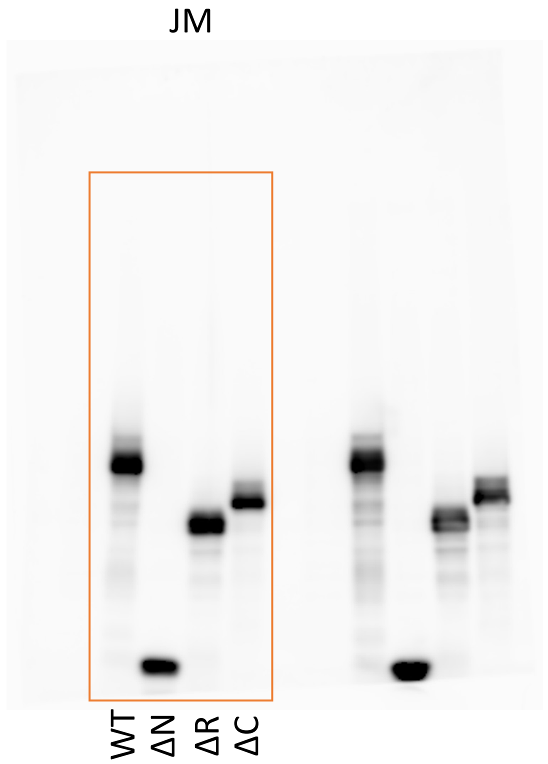


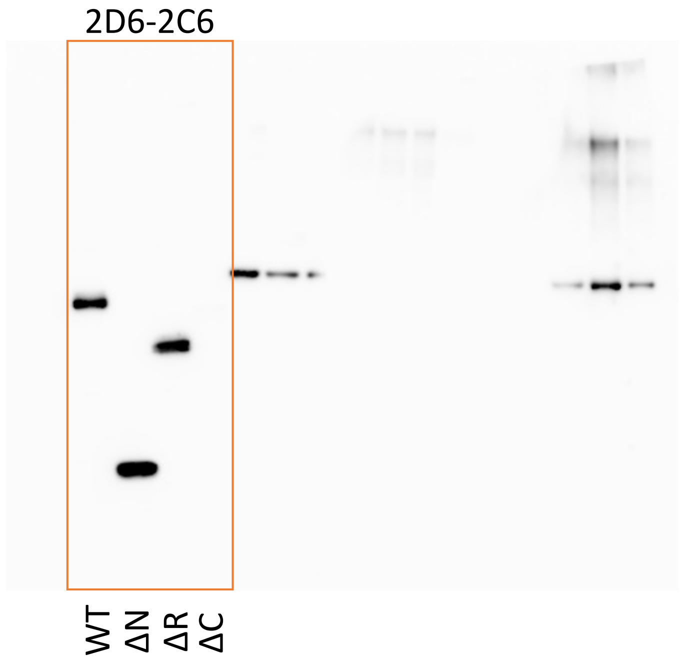


The bands placed in the Fig. 4B

**Supplemental figure 5 continued**

(J)

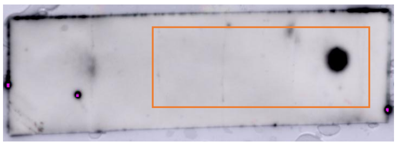

The dots placed in the Fig. 4D

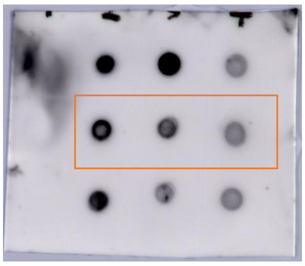

The dots placed in the Fig. 4E

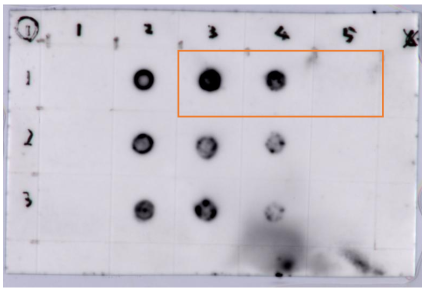

The dots placed in the Fig. 4F

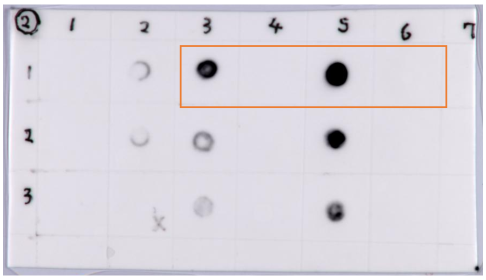

The dots placed in the Fig. 4G

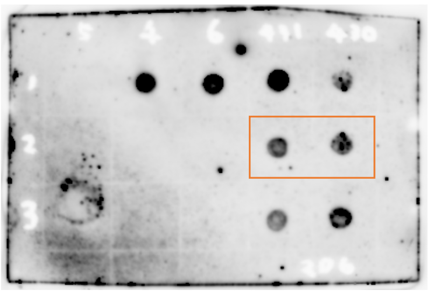

The dots placed in the Fig. 4H

**Supplemental figure 5 continued**

(K)

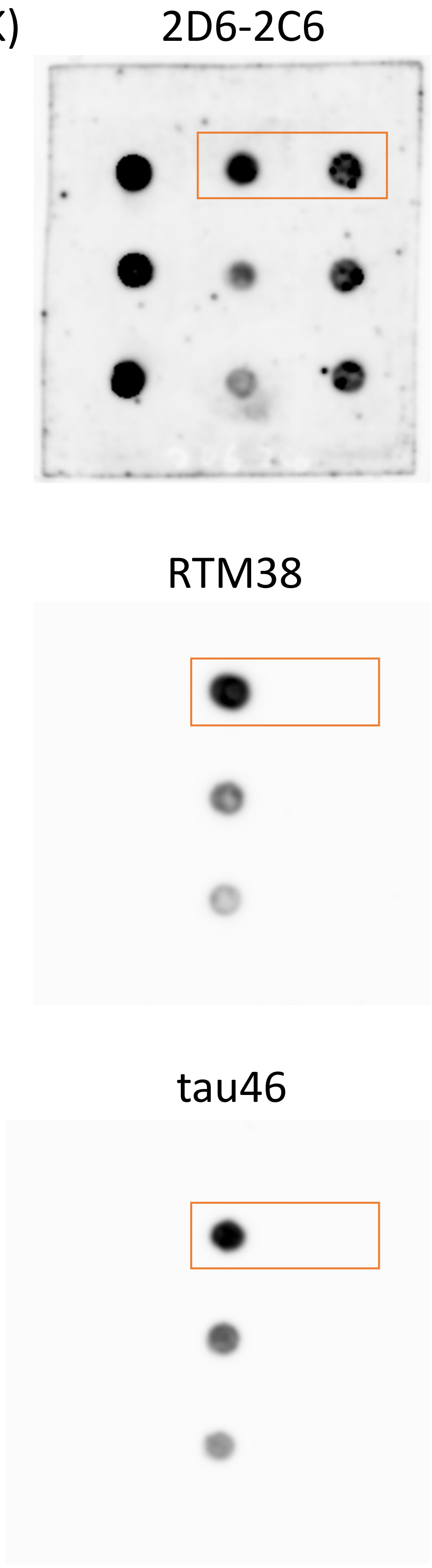


The dots placed in the Fig. 5B

**Supplemental figure 5 continued**

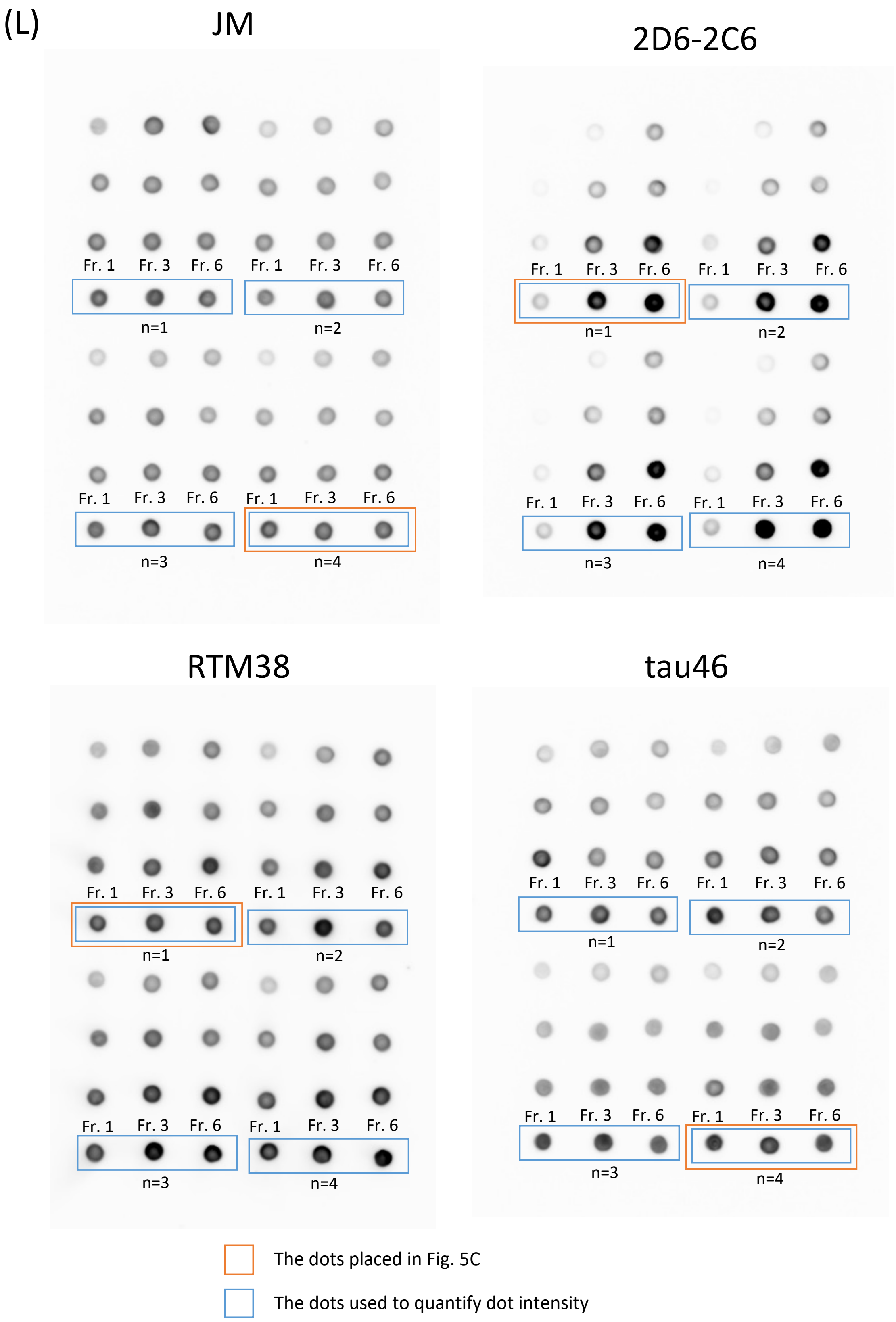

**Supplemental Figure 5: Raw data of dot blot and western blot analysis.**

(A-F) Sucrose step gradient centrifugation fractionated polymerized full-length recombinant human tau into fractions (Fr) 1-6. Tau in the fractions was detected with dot blot analysis using antibodies JM (A,F), 2D6-2C6 (B), 2B2-1B6 (C), 8D6-1F7 (D), and MC1 (E). The dotted signals enclosed in solid squares were cropped from different parts of the same membrane and are shown in Figure 1, and Supplemental Figures 2 and 4.

(G) The TBS-soluble fraction was obtained from rTg4510 mice and non-transgenic (non-Tg) mice at 10 months of age, and subjected to dot blot analysis using tau5, MC1, 2D6-2C6, 2B2-1B6 and 8D6-1F7 antibodies. The dotted signals enclosed in orange solid squares were cropped and are shown in Figures 2A-D. The dotted signals enclosed in blue solid squares were quantified and then used to create the graphs.

(H) The TBS-soluble fraction obtained from non-Tg mice was subjected to western blot analysis using 2D6-2C6 and tau5 antibodies. The bands enclosed in orange solid squares were cropped and are shown in Figure 2G.

(I) The cell lysates from COS-7 cells expressing wild-type (WT), ΔN, ΔR, and ΔC-tau were subjected to SDS-PAGE western blot using JM and 2D6-2C6 antibodies. The bands enclosed in orange solid squares were cropped and are shown in Figure 4B.

(J) The peptides with partial sequences of the C-terminal region were dotted onto a nitrocellulose membrane and then reacted with 2D6-2C6. The dotted signals enclosed in orange solid squares were cropped and are shown in Figures 4D-H.

(K) Peptides (417-441 and 423-430) were dotted onto a nitrocellulose membrane and probed with the 2D6-2C6, RTM38, and tau46 antibodies. The dotted signals enclosed in orange solid squares were cropped and are shown in Figure 5B.

(L) A dot blot analysis detected tau in Fraction (Fr.) 1, 3, and 6 using JM, 2D6-2C6, RTM38, and tau46. The dotted signals enclosed in orange solid squares were cropped and are shown in Figure 5C. The dotted signals enclosed in blue solid squares were quantified and then used to create the graphs in Figures 5D (2D6-2C6), 5E (RTM38) and 5F (tau46).

**Supplemental Table 1: Characteristics of participants.**

| | diagnosis | age at death | sex | duration (years) | NFT Braak's stage | senile plaque CERAD score |
|---|---|---|---|---|---|---|
| Case 1 | AD | 87 | male | 4 | V | C |
| Case 2 | AD | 84 | male | 3 | V | C |
| Control | pneumonia | 67 | male | | I | B |

The neuropathologic assessment of Alzheimer's disease (AD) was conducted according to National Institute on Aging–Alzheimer's Association guidelines [3].

## Supplemental reference


1 Malia, T. J. *et al.* Epitope mapping and structural basis for the recognition of phosphorylated tau by the anti-tau antibody AT8. *Proteins* **84**, 427-434, doi:10.1002/prot.24988 (2016).

2 Maeda, S. *et al.* Granular tau oligomers as intermediates of tau filaments. *Biochemistry* **46**, 3856-3861, doi:10.1021/bi061359o (2007).

3 Montine, T. J. *et al.* National Institute on Aging-Alzheimer's Association guidelines for the neuropathologic assessment of Alzheimer's disease: a practical approach. *Acta Neuropathol.* **123**, 1-11, doi:10.1007/s00401-011-0910-3 (2012).
